# Synergy between the classical and alternative pathways of complement is essential for conferring effective protection against the pandemic influenza A(H1N1) 2009 virus infection

**DOI:** 10.1371/journal.ppat.1006248

**Published:** 2017-03-16

**Authors:** Ajitanuj Rattan, Shailesh D. Pawar, Renuka Nawadkar, Neeraja Kulkarni, Girdhari Lal, Jayati Mullick, Arvind Sahu

**Affiliations:** 1 National Centre for Cell Science, S. P. Pune University Campus, Ganeshkhind, Pune, India; 2 Microbial Containment Complex, National Institute of Virology, Pune, India; St. Jude Children's Research Hospital, UNITED STATES

## Abstract

The pandemic influenza A(H1N1) 2009 virus caused significant morbidity and mortality worldwide thus necessitating the need to understand the host factors that influence its control. Previously, the complement system has been shown to provide protection during the seasonal influenza virus infection, however, the role of individual complement pathways is not yet clear. Here, we have dissected the role of intact complement as well as of its individual activation pathways during the pandemic influenza virus infection using mouse strains deficient in various complement components. We show that the virus infection in C3^-/-^ mice results in increased viral load and 100% mortality, which can be reversed by adoptive transfer of naïve wild-type (WT) splenocytes, purified splenic B cells, or passive transfer of immune sera from WT, but not C3^-/-^ mice. Blocking of C3a and/or C5a receptor signaling in WT mice using receptor antagonists and use of C3aR^-/-^ and C5aR^-/-^ mice showed significant mortality after blocking/ablation of C3aR, with little or no effect after blocking/ablation of C5aR. Intriguingly, deficiency of C4 and FB in mice resulted in only partial mortality (24%-32%) suggesting a necessary cross-talk between the classical/lectin and alternative pathways for providing effective protection. *In vitro* virus neutralization experiments performed to probe the cross-talk between the various pathways indicated that activation of the classical and alternative pathways in concert, owing to coating of viral surface by antibodies, is needed for its efficient neutralization. Examination of the virus-specific complement-binding antibodies in virus positive subjects showed that their levels vary among individuals. Together these results indicate that cooperation between the classical and alternative pathways not only result in efficient direct neutralization of the pandemic influenza virus, but also lead to the optimum generation of C3a, which when sensed by the immune cells along with the antigen culminates in generation of effective protective immune responses.

## Author summary

The pandemic influenza A(H1N1) 2009 virus is now circulating seasonally and causing a significant disease burden worldwide. Hence, it is important to delineate the immune components required for protection against its infection. Here we demonstrate that presence of intact complement is essential for clearing the pandemic influenza virus infection, wherein complement synthesized by B cells plays a major role. Further, we show that activation of the classical as well as alternative pathways is a requisite for efficient neutralization of the virus as well as the optimum generation of C3a, which is necessary for boosting the protective immune responses. Our results thus reveal that deficiencies of components of the classical and alternative pathways enhance the susceptibility to and severity of the pandemic influenza virus infection.

## Introduction

Influenza viruses, the members of the family *Orthomyxoviridae*, are globally important acute respiratory pathogens known to cause significant morbidity and mortality [1–3]. These negative sense RNA viruses possess a segmented genome encompassing a constellation of genes, which facilitate genetic reassortment upon infection with more than one influenza A virus strains [4–6]. This may result in generation of a novel virus with the ability to cause pandemics in humans [3]. One such example of a reassortant is the emergence of the novel swine-originated influenza A(H1N1) 2009 virus [A(H1N1)pdm09] that caused the first pandemic of the 21^st^ century infecting a large population and about 200,000 confirmed human deaths worldwide within the first 12 months [7–9]. The virus possesses a unique genetic combination of swine, avian and human influenza virus genes encompassing assemblage of genes such as hemagglutinin (HA), nucleoprotein (NP) and non-structural (NS) segments from the classical swine H1N1 lineage, polymerase basic 1 (PB1), polymerase basic 2 (PB2) and polymerase A (PA) from the North American H3N2 triple reassortant swine lineage, and the neuraminidase (NA) and matrix (M) segments from the Eurasian swine virus lineage [10]. The A(H1N1)pdm09 virus was first detected in India in May 2009 [11] and since then there have been outbreaks in many parts of the country. Genetic characterization of the pandemic virus isolates from India indicated that they clustered with the globally circulating prototype strain [11] with indigenous transmission in the country [12]. Within a short time the A(H1N1)pdm09 virus became one of the predominant subtypes in the country co-circulating with the seasonal influenza virus strains [13]. Genetic and antigenic characterization of this virus suggested it to be distinct from the seasonal human influenza A(H1N1) strain [14]. Thus, identification of factors influencing the pathogenesis and control of the pandemic influenza virus is essential.

The complement system is one of the critical barriers against viruses [15–17]. It has the ability to recognize and eliminate viruses directly as well as indirectly [18]. The direct mechanisms include neutralization owing to aggregation, opsonisation, lysis and promotion of phagocytosis via complement receptors [15,19], whereas the indirect mechanisms involve modulation of the adaptive immunity owing to interaction between the complement components and constituents of the adaptive immune responses [20,21]. In particular, complement has been shown to play an essential role in augmentation of virus-specific B cell and T cell responses [21–25].

Studies in the animal models have demonstrated that the complement system plays an important role in providing protection against the seasonal influenza virus infection [16,26,27], which is expected to be due to its ability to control the virus by neutralization, and enhancement of the protective immune responses. In support of this argument, the classical pathway (CP) has been shown to neutralize the seasonal influenza viruses directly with the help of natural [28] and induced antibodies [29]. And the deficiency of C3 has been shown to be associated with reduced priming of T cells in the lung-draining lymph nodes (dLN) and recruitment of effector T cells into the lung [16]. Recently, this priming defect was attributed to decreased dendritic cell (DC)-mediated viral antigen transport to the dLN, and signalling through C3aR/C5aR was found to be crucial for DC migration from the lung to dLN [27]. Which complement pathway(s) contribute towards the augmentation of these *in vivo* protective immune responses however is still not clearly understood.

The novel 2009 pandemic influenza H1N1 virus has been shown to activate complement [30], but whether complement is capable of neutralizing this virus and what role the individual complement pathways play in its neutralization, and in controlling the infection has not yet been studied. In the present study, we therefore have asked what role intact complement (using C3^-/-^ mice) and its individual complement pathways (using C4^-/-^ and factor B^-/-^ mice) play in controlling the pandemic influenza virus infection, and whether the pandemic influenza H1N1 virus is susceptible to neutralization by all the complement pathways. Our data show that deficiency of intact complement results in heightened vulnerability to the pandemic influenza virus infection in mice leading to complete mortality, and that synergy between the classical and alternative pathways is necessary for efficient protection.

## Results

### Cooperativity between the classical/lectin and alternative pathway is necessary for complete protection against the pandemic influenza virus infection in mice

**Fig 1:**
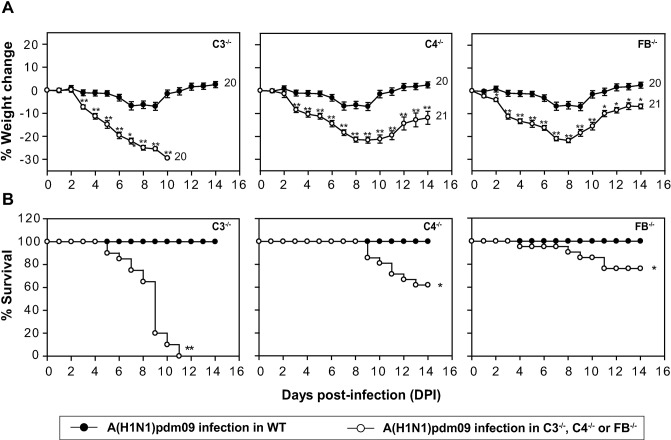
Complement deficient mice are highly susceptible to A(H1N1)pdm09 virus infection. Mice were infected intranasally with 450 TCID_50_ of the virus in PBS and monitored for body weight loss and survival for 14 days post-infection. Mock infection controls were formed by challenging the mice intranasally with normal allantoic fluid diluted in PBS. A) Percentage of body weight loss during the infection in C3^-/-^, C4^-/-^ and FB^-/-^ mice (all on C57BL/6 background) in comparison to wild-type (WT; C57BL/6) mice. Body weight was normalized to their initial body weight. The numbers at the end of the lines indicate the total number of animals utilized in each group. Weight loss was much more pronounced in C3^-/-^ mice compared to C4^-/-^ and FB^-/-^ mice. Statistical comparisons were performed between the WT mice and the complement deficient mice (C3^-/-^, C4^-/-^ and FB^-/-^). *p < 0.01 and **p < 0.001 (Mann–Whitney Rank Sum test, IBM PASW). B) Percentage of survival during the infection in C3^-/-^, C4^-/-^ and FB^-/-^ mice (all on C57BL/6 background) in comparison to WT (C57BL/6) mice. The survival in C3^-/-^, C4^-/-^ and FB^-/-^ mice was significantly lower than the WT mice (*p < 0.02; **p < 0.001). C4^-/-^ and FB^-/-^ mice showed partial mortality, while C3^-/-^ mice showed complete mortality. Results represent mean ± SEM of three independent experiments.

The role of the individual complement pathways during influenza virus infection is not clear. Thus to address this, we examined the relative *in vivo* contribution of the individual complement pathways in providing protection against the A(H1N1)pdm09 virus infection. All the three pathways converge at C3 activation step. Hence to understand the role of intact complement, C3^-/-^ mice were infected by inoculating a sub-lethal dose of the virus by the intranasal route. Infection in C3^-/-^ mice showed severe illness with significant weight loss leading to 100% mortality by day 11 post-infection (p.i.) **(Fig 1A & 1B)**. However, WT mice showed only 10% weight loss at the peak of infection, and all mice fully recovered at day 12 p.i (**Fig 1A & 1B**), strongly establishing that complement plays a protective role during the pandemic influenza virus infection.

Next, to determine the contribution of the individual pathways, we infected C4^-/-^ mice [deficient in classical pathway (CP)/lectin pathway (LP)] and FB^-/-^ mice (deficient in alternative pathway; AP) and monitored them for weight loss and mortality. Results showed significant weight loss in both the knockout strains compared to the WT mice (**Fig 1A**) with 32% mortality in C4^-/-^ and 24% mortality in FB^-/-^ mice (**Fig 1B)**. Together, these data suggest that the CP/LP and AP are capable of providing a certain degree of protection owing to the activation of each in the absence of the other, however, cooperativity between these pathways is needed to provide complete protection against the influenza A(H1N1)pdm09 virus infection.

### Complement-deficient mice show enhanced pulmonary pathology and delayed virus clearance

**Fig 2:**
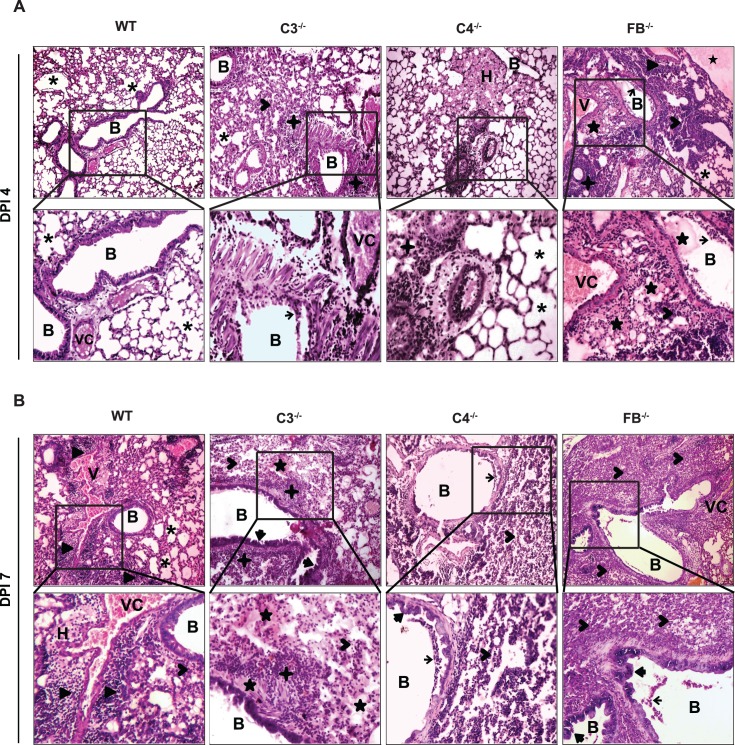
Complement deficient mice infected with A(H1N1)pdm09 virus show increased lung inflammation. WT (C57BL/6) and complement deficient mice (C3^-/-^, C4^-/-^ and FB^-/-^ mice on C57BL/6 background) were infected intranasally with the pandemic influenza virus (450 TCID_50_), euthanized at day 4 (*panel A*) or 7 (*panel B*) post-infection, and lungs were harvested for histopathology. Sections were stained with H & E and are representative of each group (n = 6, in each group). Magnified view (400X) of the marked area for each panels are displayed in the bottom rows. C3 and FB deficiency was associated with increased inflammatory changes compared to C4 deficiency. Typical histopathological changes include: Emphysema (asterisk), Edema (star), Peri-bronchial (plus) and peri-vascular (filled arrowhead) lymphoid cell infiltrate, parenchymal MNC infiltration (unfilled arrowhead), Hyperplasia (filled arrow), and Sloughing (unfilled arrow). B = bronchi, H = hemorrhage, V = blood vessel, VC = vascular congestion. Original magnification = 100X.

To determine whether the increased mortality observed above in the complement deficient mice infected with the A(H1N1)pdm09 virus is associated with increased pulmonary pathology in these mice (C3^-/-^, C4^-/-^ and FB^-/-^), we performed histopathological analysis of the lungs collected at days 4 and 7 p.i. Mock infected mice showed normal lung architecture with intact cellular details of alveoli, bronchi and blood vessels at both these time points (S1 **Fig**). At day 4 p.i. (**Fig 2A**), virus infected WT mice showed minimal pathological changes with congested blood vessels and mild emphysematous changes of alveolar parenchyma and normal bronchiolar epithelium. However, at day 7 p.i. (**Fig 2B**), the virus infected WT mice showed mild-to-moderate pathological changes that included foci of haemorrhage in lung parenchyma/alveoli, peri-bronchial and peri-vascular infiltration of inflammatory mononuclear cells (MNCs) comprising mainly of lymphocytes, alveolar macrophages (AMs), neutrophils and few histiocytes. Other changes include exudation, emphysema, and interstitial deposits of inflammatory cells with alveolitis. The histopathological changes at both the time points were also apparent in C3^-/-^ and FB^-/-^ infected mice, but the overall degree of infiltration of inflammatory MNCs was higher in these mice compared to the WT mice. In addition, multiple foci of degenerative changes including loss of bronchiolar epithelium (sloughing) and bronchiolar hyperplasia, with edematous changes and consolidation of alveolar parenchyma were also observed in these mice. The C4^-/-^ mice also showed similar changes, but the degree of inflammation was more similar to the WT mice (**Fig 2A & 2B**).

**Fig 3:**
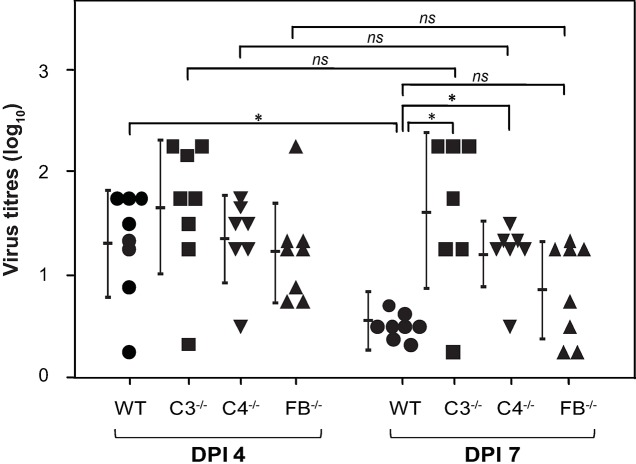
Complement deficient mice infected with A(H1N1)pdm09 virus show prolonged infection. WT (C57BL/6) and complement deficient mice (C3^-/-^, C4^-/-^ and FB^-/-^ mice on C57BL/6 background) were infected intranasally with the pandemic influenza virus (450 TCID_50_), euthanized at day 4 or 7 post-infection, and lungs were harvested for estimation of the virus load. Virus titer was determined in the lung homogenates by TCID_50_ analysis. C3^-/-^ and C4^-/-^ mice retained significantly higher virus load at day 7 post-infection compared to the WT mice. Bars represent mean ± SD. *p < 0.05 (Mann–Whitney Rank Sum test, IBM PASW). *ns* indicates not significant.

We next assessed the presence of infectious virus in the lungs of C3^-/-^, C4^-/-^ and FB^-/-^ mice infected with A(H1N1)pdm09 virus and compared that to the infectious virus titer in the lungs of infected WT mice (**Fig 3**). The viral load was found to be higher during the initial phase of the infection i.e., at day 4 p.i., in complement deficient (C3^-/-^, C4^-/-^ and FB^-/-^) as well as WT mice. The load however decreased significantly at day 7 p.i. in the WT mice (p < 0.012), but not in the C3^-/-^ mice suggesting that these mice are unable to clear the virus efficiently. The infectious virus titer also remained high in the C4^-/-^ and FB^-/-^ mice, but was relatively lower compared to that found in the C3^-/-^ mice **(Fig 3).** Collectively, these data suggest that individual complement pathways are capable of clearing the virus to a certain extent, but their summative association is required for the efficient clearance.

### C3^-/-^ and C4^-/-^ mice, but not FB^-/-^ mice, show reduced antibody response to pandemic influenza virus hemagglutinin

**Fig 4:**
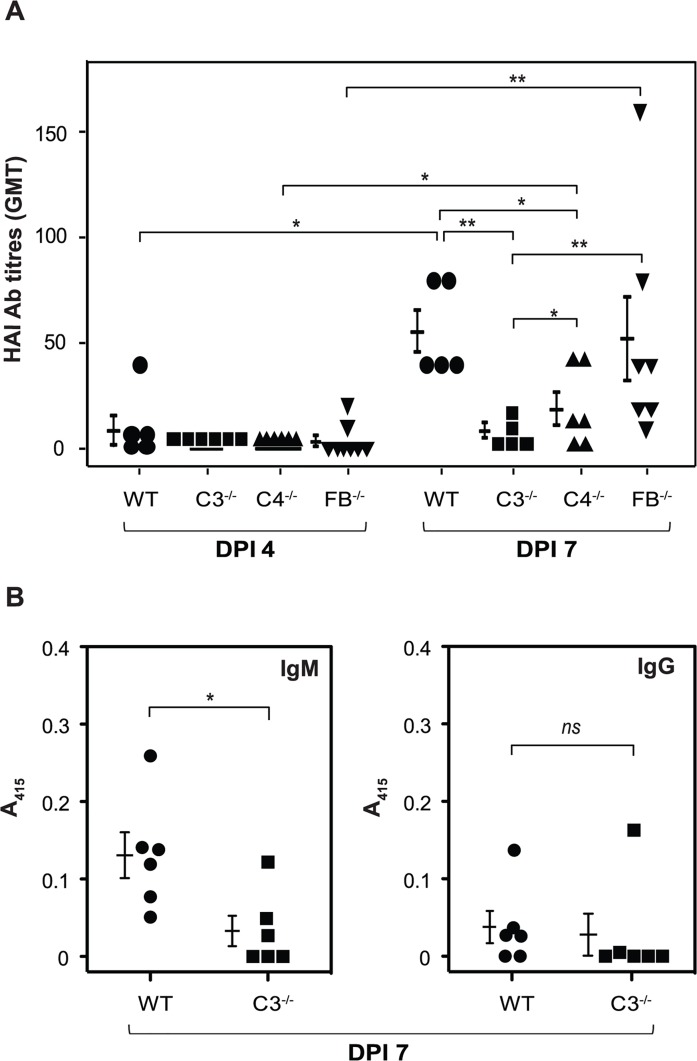
Antibody response to hemagglutinin is significantly reduced in pandemic influenza virus infected C3^-/-^ and C4^-/-^ mice, but not FB^-/-^ mice. WT (C57BL/6) and complement deficient mice (C3^-/-^, C4^-/-^ and FB^-/-^ mice on C57BL/6 background) were infected intranasally with the pandemic influenza virus (450 TCID_50_), euthanized at day 4 and 7 post-infection, and serum was collected to determine the HA-specific antibody response. A) HAI antibody titers. Data are plotted as Geometric mean titers (GMT). Bars represent mean ± SD of three independent experiments. *p < 0.05 and **p < 0.002 (Mann–Whitney Rank Sum test, IBM PASW). B) HA-specific IgM and IgG levels as measured by ELISA. Bars represent mean ± SEM. *p < 0.02; *ns*—not significant (Mann–Whitney Rank Sum test, IBM PASW).

Development of the antibody response against influenza hemagglutinin (HA) is linked with immune protection [31]. Thus, next we asked whether the antibody response to HA is reduced in the pandemic influenza virus infected complement deficient mice (C3^-/-^, C4^-/-^ and FB^-/-^) compared to the WT mice. Examination of the HA-inhibition (HAI) antibody titers in the WT mice showed lower titers at day 4 p.i., and this significantly increased at day 7 p.i. (p < 0.05, **Fig 4A**). This marked increase in the HAI antibody titer however was not observed in C3^-/-^ and C4^-/-^ mice, suggesting that the loss of immune protection in these mice could, in part, be due to decrease in the antibody titer. This raised the question: Are these protective antibodies IgM or IgG? Measurement of the pandemic influenza A virus HA-specific IgM and IgG in sera of WT and C3^-/-^ mice 7 days p.i. showed that IgM, but not IgG levels are significantly lower in C3^-/-^ mice compared to the WT mice (**Fig 4B)**. Consequently, protection seems to be primarily mediated by IgM. Interestingly, the HAI antibody titers in FB^-/-^ mice at day 7 p.i. were similar to those in the WT mice, suggesting that immune factors other than the HAI antibodies ought to be responsible for the partial loss in immune protection seen in these mice (**Fig 1B**).

### Passive administration of immune sera from WT mice, but not from C3^-/-^ mice, provide protection against the pandemic influenza virus infection in C3^-/-^ mice

**Fig 5:**
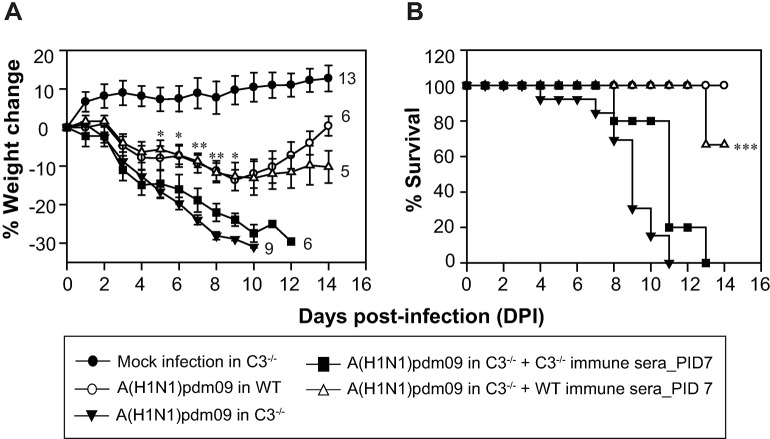
Antibodies generated during early phase of the infection are critical in containing the virus infection. C3^-/-^ mice on C57BL/6 background, untreated or treated with immune sera (250μl each at day -1 and 6 post-infection), were infected intranasally with 450 TCID_50_ of the virus in PBS and monitored for body weight loss and survival for 14 days post-infection. A) Percentage of body weight loss during the infection in mice untreated or treated with the indicated sera. Body weight was normalized to their initial body weight. The number of animals utilized in each group is shown at the end of the line for that group. Statistical comparisons were performed between C3^-/-^ immune sera treated and WT immune sera treated mice. Significant weight gain was observed in WT immune sera treated mice. *p < 0.05 and **p < 0.02. B) Percentage of survival during the infection in mice untreated or treated with the indicated sera. About 67% of the mice treated with WT immune sera recovered. C3^-/-^ immune sera treated versus WT immune sera treated mice: ***p < 0.001.

Because C3^-/-^ mice showed significantly decreased HAI antibody titer compared to the WT mice, we determined whether the loss of protection against the influenza A(H1N1)pdm09 virus infection in C3^-/-^ mice is indeed, in part, due to the decreased antibody response. Thus, immune sera collected at day 7 p.i. from the WT or C3^-/-^ mice was heat-inactivated, and injected intraperitoneally (i.p.) in naïve C3^-/-^ mice, which were then challenged by intranasal (i.n.) route with the pandemic influenza virus. The immune sera from the WT mice significantly prevented the weight loss **(Fig 5A)** and mortality **(Fig 5B)**. However, the immune sera from the C3^-/-^ mice failed to prevent the weight loss and only slightly delayed mortality **(Fig 5).** Together, these results suggest that impaired virus-specific antibody response, in part, is responsible for the severe viral infection and mortality in C3^-/-^ mice.

### Adoptive transfer of WT splenocytes or splenic B cells into C3^-/-^ mice restores protection against the pandemic influenza virus infection

**Fig 6:**
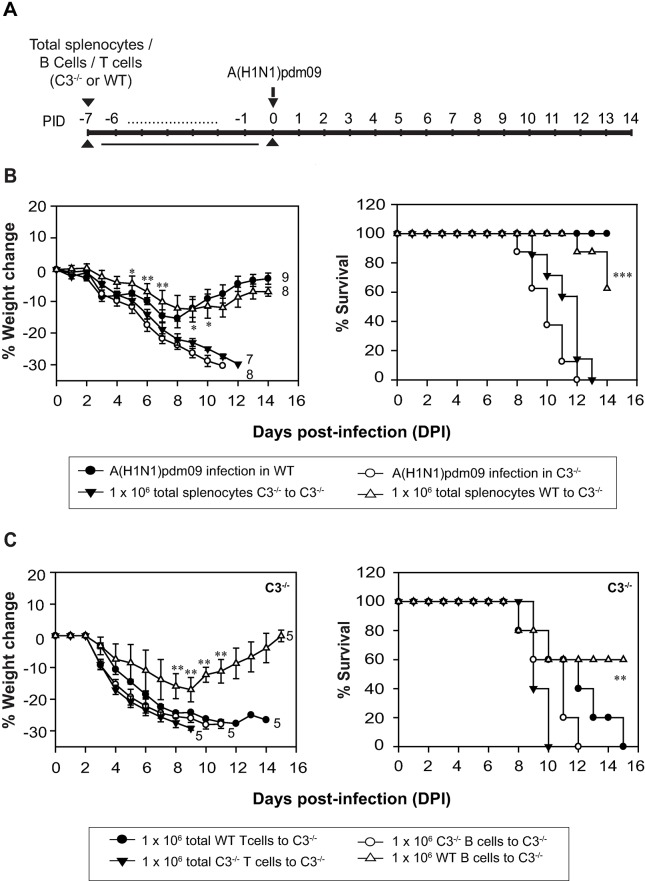
Adoptive transfer of WT splenocytes or purified B cells to C3^-/-^ mice restores protection against the pandemic influenza virus infection. A) Schematic diagram showing the experimental setup. C3^-/-^ mice on C57BL/6 background were injected with 1 X 10^6^ splenocytes or purified T cells or B cells (WT or C3^-/-^) via tail vein at day minus 7 pre-infection. Thereafter, they were infected intranasally with 450 TCID_50_ of the virus and monitored for body weight loss and survival for 14 days post-infection. Control mice did not receive splenocytes. B) Adoptive transfer of splenocytes. *Left panel*, the percentage of body weight loss during the infection in mice receiving WT or C3^-/-^ splenocytes. Body weight was normalized to their initial weight. The number of animals utilized in each group is shown at the end of the line for that group. WT splenocytes to C3^-/-^ mice versus C3^-/-^ splenocytes to C3^-/-^ mice: *p < 0.05 and **p < 0.02. *Right panel*, the percentage of survival during the infection in C3^-/-^ mice receiving WT or C3^-/-^ splenocytes. Results represent mean ± SEM of two independent experiments. WT splenocytes to C3^-/-^ mice versus C3^-/-^ splenocytes to C3^-/-^ mice: ***p < 0.001. C) Adoptive transfer of purified splenic T or B cells. *Left panel*, percentage of body weight loss during the infection in C3^-/-^ mice receiving either T or B cells (WT or C3^-/-^). Body weight was normalized to their initial weight. The number of animals utilized in each group is shown at the end of the line for that group. WT B cells to C3^-/-^ mice versus C3^-/-^ B cells to C3^-/-^ mice: **p < 0.02. *Right panel*, percentage of survival during the infection in C3^-/-^ mice receiving either T cells or B cells (WT or C3^-/-^). WT B cells to C3^-/-^ mice versus C3^-/-^ B cells to C3^-/-^ mice: **p < 0.02.

It was clear from the above data that C3 plays a pivotal role in enhancing the protective humoral response against the pandemic influenza virus infection. The major source of C3 is liver, but extra-hepatic C3 has been shown to play a key role in enhancing the humoral response to peripheral viral infection [32]. Therefore, this raised the question whether liver-derived C3 or extra-hepatic C3 is more critical in providing the protection. To distinguish the contribution of C3 produced from liver cells and leukocytes, we adoptively transferred splenocytes either from naïve WT or from naïve C3^-/-^ mice into C3^-/-^ mice, and challenged these mice with the A(H1N1)pdm09 virus (**Fig 6A**). Results showed that the C3^-/-^ mice that received WT splenocytes showed recovery in weight and increased survival (62.5%; p < 0.001) whereas those that received C3^-/-^ splenocytes failed to prevent weight loss and caused complete mortality **(Fig 6B)**. These data therefore point out a major role of extra-hepatic C3 in providing protection against the A(H1N1)pdm09 virus. Next, we determined whether splenic T cells or B cells can serve as a source of C3 and correct the C3^-/-^ phenotype as both are known to synthesize C3 [33]. We thus purified T and B cells from the spleen of naïve WT or C3^-/-^ mice (**S3 Fig**) and adoptively transferred into C3^-/-^ mice which were then challenged with the A(H1N1)pdm09 virus (**Fig 6C**). We observed that WT B cells, but not WT T cells could partially protect the C3^-/-^ mice against the pandemic influenza virus infection suggesting that C3 produced by B cells contribute significantly to the generation of protective immune response against the virus.

### C3a-mediated signaling is crucial for generation of the protective immune response against the pandemic influenza virus in mice

**Fig 7:**
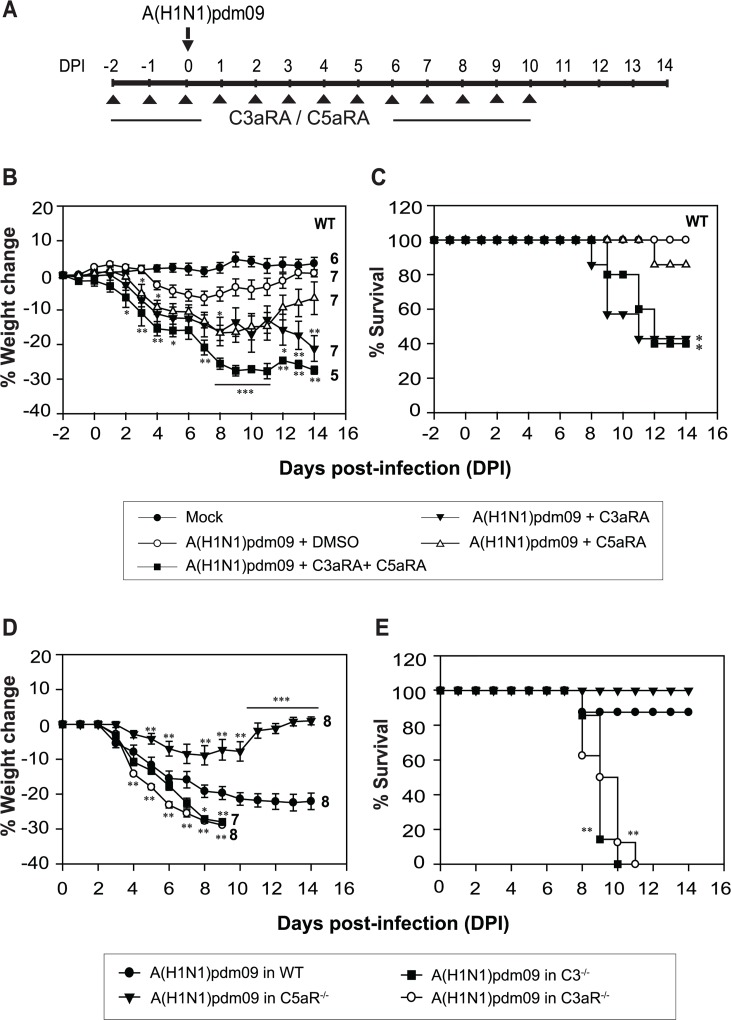
C3a signaling is primarily important for generating protective immune response against the A(H1N1)pdm09 virus. A) Schematic diagram showing the experimental setup. WT (C57BL/6) mice administered with C3aRA or C5aRA or both (1mg/kg of body weight through i.p. route) were infected intranasally with 450 TCID_50_ of the virus in PBS and monitored for weight loss and survival for 14 days post-infection. Arrow indicates the day of infection and arrowheads indicate the days on which C3aRA and/or C5aRA was injected. B) Percentage of body weight loss during infection in indicated mice groups; numbers at the end of the lines indicate the number of animals. Significant weight loss was observed in all the receptor antagonist treated groups compared to untreated group (*p <0.05, **p < 0.002 and ***p < 0.0001). C) Percentage of survival during the infection in indicated mice groups. The survival in C3aRA and C3aRA/C5aRA treated mice was significantly lower than in WT mice (*p <0.02). Results represent mean ± SEM of two independent experiments. D) Percentage of body weight loss during the infection in C3aR^-/-^, C5aR^-/-^ and C3^-/-^ mice (all on BALB/c background) in comparison to WT (BALB/c) mice. Results represent mean ± SEM. (*p <0.05, **p < 0.02 and ***p < 0.001). E) Percentage of survival during the infection in C3aR^-/-^, C5aR^-/-^ and C3^-/-^ mice on BALB/c background in comparison to WT (BALB/c) mice. The C3aR^-/-^ and C3^-/-^ mice showed complete mortality, while WT and C5aR^-/-^ mice showed little or no mortality (**p ≤ 0.002).

Complement activation results in the generation of immuno-modulatory peptides termed C3a and C5a. These peptides have been shown to modulate the expression of costimulatory molecules on dendritic cells by signaling through their receptors C3aR and C5aR and thereby influence T-cell priming [34]. Their role has also been studied in seasonal influenza virus infection. Signaling through C3aR and C5aR has been shown to be critical for migration of dendritic cells to the draining lymph node in response to influenza virus infection [27]. In view of the above, we asked, is C3aR and C5aR signaling vital during the pandemic influenza virus infection? We thus blocked the C3aR and/or C5aR signalling in the WT mice by pharmacologically targeting these receptors with specific antagonists (C3aRA and C5aRA), and monitored the severity of infection. Thus, WT mice challenged with A(H1N1)pdm09 virus received a daily i.p. injection of C3aRA or C5aRA or both (1mg/kg of body weight) from day -2 to 10 p.i. **(Fig 7A)**. Results showed significant weight loss and mortality (60%) in mice that received both C3aRA and C5aRA compared to the vehicle treated mice. Mice treated with C5aRA alone however recovered well with only 10% mortality. Notably, mice that received only C3aRA also showed significant weight loss with 57% mortality (**Fig 7B & 7C**). These data suggest that C3aR-mediated signalling plays a predominant role in generating a protective immune response to the pandemic influenza virus, with signalling through C5aR playing a relatively minor role.

To further strengthen our conclusion that C3aR plays a principal role during pandemic influenza virus infection, we also performed infection experiment in C3aR^-/-^ and C5aR^-/-^ mice available on BALB/c background. Although earlier studies have shown that A(H1N1)pdm09 virus infects C57BL/6 and BALB/c mice equally well [35], we included C3^-/-^ mice on BALB/c background to ascertain whether mouse strain differences affect the susceptibility to the virus infection. As expected, the C3^-/-^ mice displayed sustained weight loss with 100% mortality; only 10% mortality was observed in WT BALB/c mice (**Fig 7D and 7E**). Further, conforming to our receptor antagonist data (**Fig 7B & 7C**), C3aR^-/-^ mice showed heightened susceptibility to the pandemic influenza virus infection, while C5aR^-/-^ mice showed complete recovery. If anything, C5aR^-/-^ mice recovered better than the WT mice.

### Pandemic influenza virus is susceptible to neutralization by mouse complement only when coated by antibody

**Fig 8:**
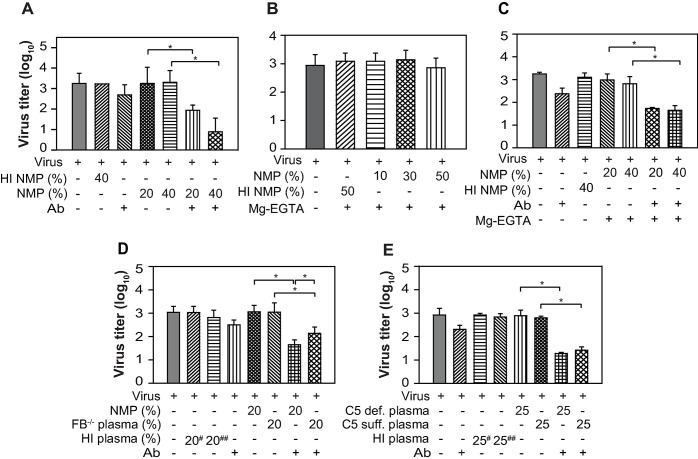
A(H1N1)pdm09 virus is susceptible to the classical pathway, but resistant to the alternative pathway-mediated neutralization unless coated by antibodies. A) CP-mediated neutralization of A(H1N1)pdm09 virus. The virus was incubated with normal mouse plasma (NMP; 20% & 40%; B6 mice) in the presence or absence of antibody, and the infectious virus remained was determined by TCID_50_. B) AP-mediated neutralization of the virus. The virus was incubated with the various concentrations of normal mouse plasma (10%, 30% or 50%; B6 mice) in the presence of 20 mM Mg-EGTA and the remaining virus was titrated by TCID_50_. C) AP-mediated neutralization of A(H1N1)pdm09 virus in the presence of virus specific antibodies. The virus was incubated with two different concentrations of normal mouse plasma (20% and 40%; B6 mice) in the presence of antibody and Mg-EGTA, and the remaining virus was titrated by TCID_50_. D) Contribution of the AP amplification loop during the CP-mediated neutralization of A(H1N1)pdm09 virus. The virus was incubated with WT or FB^-/-^ mouse plasma (20%; B6 mice) in the presence of antibody and Ca^++^ and Mg^++^, and the virus remained was determined by TCID_50_ analysis. (^#^ HI—NMP; ^##^ HI—FB deficient plasma). E) A(H1N1)pdm09 virus neutralization by C5 deficient mice plasma. The virus was incubated with C5 sufficient (25%; B10.D2/nSnJ) or C5 deficient (25%; B10.D2/oSnJ) mouse plasma in the presence of antibody and Ca^++^ and Mg^++^, and the remaining virus was titrated by TCID_50_. (^#^ HI—nSn J; ^##^ HI—oSnJ plasma). Results represent mean ± SD of three independent experiments. *p ≤ 0.05.

Our *in vivo* data clearly established the role of complement in controlling the pandemic influenza virus infection in mice. Thus, next to determine whether the virus is susceptible to direct neutralization by complement we performed *in vitro* virus neutralization assays. To measure the classical pathway (CP)-mediated neutralization, the virus was incubated with normal mouse plasma (as a source of complement) in the presence of virus-specific mouse antibodies and Ca^++^-Mg^++^. And to examine the alternative pathway (AP)-mediated neutralization, the virus was incubated with normal mouse plasma in the presence of Mg-EGTA which allows selective activation of the AP. Our results showed that the virus was efficiently neutralized by CP (**Fig 8A**), but not by AP (**Fig 8B**). Though these results were in agreement with enhanced infection seen in C4^-/-^ mice, they were in sharp contrast to the enhanced infection observed in FB^-/-^ mice (**Fig 1**). We thus tested the possibility whether the pandemic virus becomes susceptible to AP-mediated neutralization when coated by antibodies. It is clear from the results presented in **Fig 8C** that the virus indeed becomes susceptible to neutralization by AP when coated by antibodies. Together these results suggest that the virus becomes susceptible to complement either by CP or by AP only when coated by antibodies.

We observed *in vivo* synergy between the CP and AP of complement for providing protection against the pandemic influenza virus infection. To address whether such synergy also operates during *in vitro* neutralization of the virus, we tested if the CP-mediated deposition of C3b onto the viral surface leads to activation of the AP loop and thereby augment neutralization. Examination of the CP-mediated neutralization of the virus using FB^-/-^ mouse plasma showed significant reduction in the extent of neutralization compared to the normal mouse plasma (**Fig 8D**) suggesting that synergy does exist between the classical and alternative pathways during the pandemic virus neutralization.

Earlier studies on seasonal influenza virus have demonstrated that deposition of the early complement component (e.g., C3b and C4b) followed by aggregation of the virus is enough for neutralization, and activation of the terminal pathway leading to MAC-mediated lysis is not essential [28]. We thus also looked into the requirement of the terminal pathway for CP-mediated neutralization of the pandemic influenza virus. We observed that C5-deficient plasma was able to neutralize the virus as efficiently as the C5-sufficient plasma (**Fig 8E**). It is thus evident that MAC-mediated lysis is not necessary for the neutralization of the pandemic influenza virus.

### Pandemic influenza virus is also efficiently neutralized by human complement in the presence of antibody

**Fig 9:**
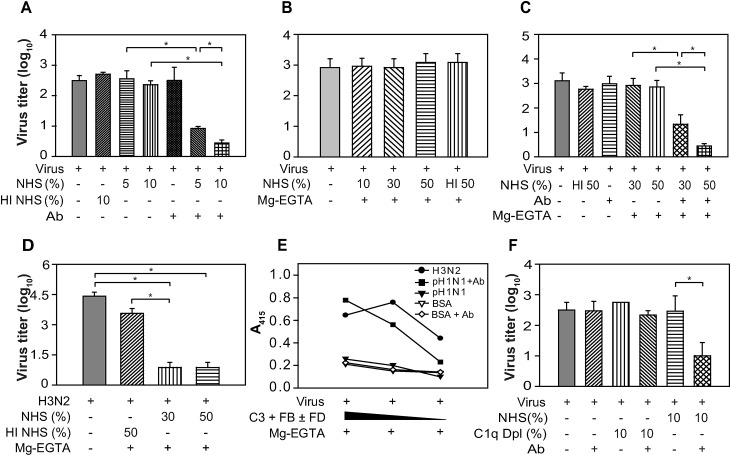
A(H1N1)pdm09 virus neutralization by human complement. A) CP-mediated neutralization of A(H1N1)pdm09 virus by NHS in the presence or absence of antibody. B) AP-mediated neutralization of A(H1N1)pdm09 virus by NHS in the presence of Mg-EGTA. C) Alternative pathway-mediated neutralization of A(H1N1)pdm09 virus in the presence of antibody and Mg-EGTA. D) AP-mediated neutralization of H3N2-A/Perth virus by NHS in the presence of Mg-EGTA. E) AP-mediated C3b deposition on H3N2-A/Perth09, and A(H1N1)pdm09 viruses in the presence or absence of antibody. F) A(H1N1)pdm09 virus neutralization by C1q depleted sera in the presence or absence of antibody. All the neutralization experiments were performed in an essentially similar manner to that performed with mouse complement. Results represent mean ± SD for three independent experiments except for panel E, which shows mean of two independent experiments. *p ≤ 0.05.

Influenza A(H1N1)pdm09 virus is a human pathogen, hence next we sought to establish the susceptibility of this virus to the human CP- and AP-mediated neutralization. The neutralization assays employed were essentially similar to those described above for the mouse complement, except that the human sera (a source of complement) and virus-specific human antibodies (isolated from the pandemic influenza virus infected subjects) were used. The results obtained were essentially similar to that observed using the mouse complement: the virus showed susceptibility to CP-mediated neutralization, but resistance to AP-mediated neutralization **(Fig 9A & 9B)**. And as expected, coating of virus with antibody resulted in AP-mediated neutralization (**Fig 9C**).

We next asked whether seasonal influenza A virus, A/Perth/16/2009(H3N2), is also resistant to AP-mediated neutralization. Our results showed that unlike the pandemic influenza virus, seasonal influenza virus is susceptible to AP-mediated neutralization (**Fig 9D**). These results intrigued us to investigate whether the difference in AP-mediated neutralization of the above two viruses is due to the difference in the ability of their surfaces to allow C3b deposition. Activation of C3 near their surfaces using purified complement components confirmed our presumption: C3b deposition was efficient on the H3N2 viral surface, but not on the pandemic influenza virus unless coated by antibodies (**Fig 9E**). Thus, there exists distinct difference in the susceptibility of these two viruses towards AP-mediated neutralization.

Earlier mannose-binding lectin (MBL) has been shown to bind to various strains of the seasonal influenza viruses [36], but the LP does not seem to neutralize these viruses [28]. Therefore, we also examined if the pandemic influenza virus is susceptible to the LP-mediated neutralization using C1q-deficient human serum that lacks CP, but contains intact LP. Interestingly, C1q-deficient sera failed to neutralize the virus suggesting that LP does not play a role in neutralizing the pandemic influenza virus (**Fig 9F**).

### Pandemic influenza virus-specific complement-binding antibody response differs in different individuals

**Fig 10:**
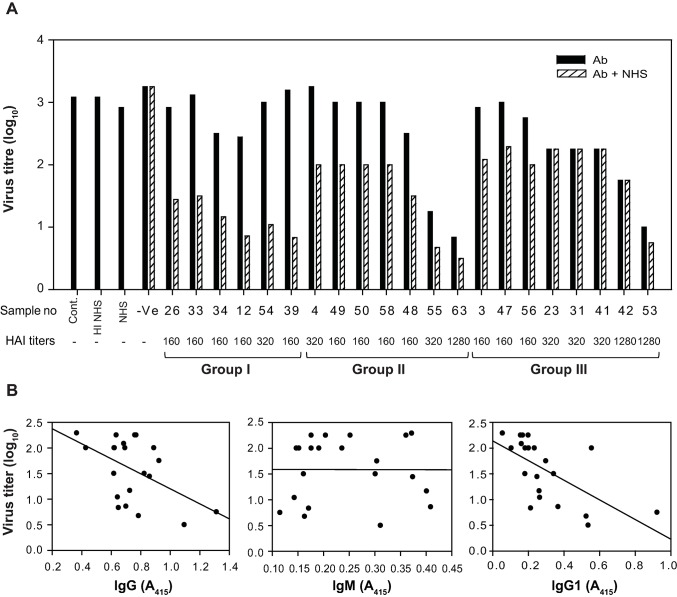
Influenza-specific complement-binding antibody response differs in different individuals. A) A total of 21 A(H1N1)pdm09 virus positive human sera were screened for the presence of complement binding antibodies. The virus was incubated with heat inactivated human sera sample collected from the A(H1N1)pdm09 virus infected individuals [HAI titer: 160–1280] in the presence or absence of normal human serum as a source of complement, and the infectious virus remained was titered by TCID_50_. Results represent mean of two independent experiments. Virus neutralization potential of serum antibodies in the presence of complement varied in different individuals. Based on the neutralization potential, sera are grouped as: Group I—substantial enhancement in neutralization in the presence of complement. Group II—moderate enhancement in neutralization in the presence of complement. Group III–negligible enhancement in neutralization in the presence of complement. B) Correlation between the virus-specific antibody levels in sera and the ability of the sera to neutralize the virus in the presence of complement. The A_415_ values are shown for comparison of antibody levels within the subclass of immunoglobulins. Significant correlation was observed only between neutralization potential of sera in the presence of complement and IgG (r = -0.501; p = 0.02) and its subclass IgG1 (r = -0.628; p < 0.002).

The observed susceptibility of the A(H1N1)pdm09 virus to the human CP portrayed the importance of the virus-specific complement-binding antibodies in controlling the virus. This raised the question whether such antibody response is robust in humans during the pandemic influenza virus infection. We therefore looked for generation of such antibodies in the infected individuals. A total of 21 human serum samples collected from the individuals, which were positive for the presence of antibodies against the A(H1N1)pdm09 virus, were heat-inactivated and screened for the presence of complement binding antibodies by measuring their ability to enhance virus neutralization in the presence of complement. The sera from the infected individuals demonstrated varied antibody-mediated complement-enhanced neutralization potential (**Fig 10A**). Based on the results, the sera were grouped as: i) showing substantial antibody-mediated complement-enhanced neutralization (>50%; Group I), ii) showing moderate antibody-mediated complement-enhanced neutralization (30%-50%; Group II), and iii) showing negligible antibody-mediated complement-enhanced neutralization (<30%; Group III) (**Fig 10A**).

Next, to determine which class/subclass of the virus-specific antibody is responsible for supporting the complement-enhanced neutralization of the virus, we looked at the dependence of antibody-mediated complement-enhanced neutralization on the class/subclass of pandemic influenza virus-specific antibody **(S2 Fig)**. We observed a significant negative correlation between the IgG titer and the virus titer that remained after the neutralization (r = - 0.501, p = 0.02) and between the IgG1 titer and the virus titer that remained after the neutralization (r = -0.628; p < 0.002). These correlations suggest that IgG1 antibodies present in the sera of A(H1N1)pdm09 virus-positive individuals are capable of supporting the complement-enhanced neutralization (**Figs 10B & S2)**.

## Discussion

The complement system has been shown to impact the control of influenza virus infections [16,27,28], though the contribution of all the respective pathways are not yet fully determined. In the present study, we have investigated which complement pathways are triggered by the influenza A(H1N1)pdm09 virus and their impact in controlling the infection. Our data establish that synergy between the CP and AP is the key to effective control of the pandemic influenza virus infection. We demonstrate that C3 deficiency results in complete lethality, while C4 or FB deficiency results only in partial lethality (24% - 32%), suggesting *in vivo* cross-talk between the CP and AP. Such cross-talk was also apparent during *in vitro* virus neutralization wherein triggering of the AP loop following the activation of CP resulted in more efficient neutralization of the virus.

Complement deficiencies (C3^-/-^, C4^-/-^ or FB^-/-^) in mice resulted in increased susceptibility to the pandemic influenza virus infection leading to partial or complete lethality. To look into the possible cause, we examined the effect of the deficiencies on the histopathological changes and viral clearance in the lung. We observed an overall higher degree of inflammation in C3^-/-^ and FB^-/-^ mice compared to the WT mice, but not in C4^-/-^ mice i.e., the lethality in mice did not correlate well with exacerbated inflammatory changes. The viral clearance in the lung however showed a better correlation with lethality: WT mice effectively cleared the virus from the lung by day 7 p.i., but the same was not observed in C3^-/-^ mice. The viral load also remained high in C4^-/-^ and FB^-/-^ mice lungs, but was lower compared to that observed in C3^-/-^ mice. Intriguingly, initial virus load did not differ between the WT and complement deficient mice suggesting that complement-mediated protection seen in WT mice was a late phenomenon which occurred after day 5 p.i.

It is now well established that there is generation of strain-specific antibody response to the surface antigens of influenza viruses such as HA [37] and NA [38] during infection. It is also known that antibodies to HA in particular are key to protection against influenza [39,40] and these HA specific antibodies bind to complement [41]. Additionally, it is also documented that these antibodies are generated about 5 days post-infection [41]. We thus asked whether generation of antibodies against HA antigen is affected in complement deficient mice, and whether individual pathways differ in inducing anti-HA response. Our results demonstrate that C3 deficiency results in a significant decrease in HA-specific antibodies (particularly of IgM subclass) suggesting that generation of HA antibodies can be modulated by complement. It is also notable that apart from C3^-/-^ mice, reduced HA antibody response was also observed in C4^-/-^, but not in FB^-/-^ mice, suggesting that the CP/LP, but not the AP is critical for generation of HA response. Similar pathway-specific difference in the antibody responses was observed earlier during West Nile virus infection [42].

Though the above data showed a correlation between the C3 deficiency and reduction in response to HA, it did not establish the causal relationship between reduction in response to HA and higher lethality in C3^-/-^ mice. Thus, to establish such a relationship, we performed rescue experiment with immune sera from A(H1N1)pdm09 virus infected WT and C3^-/-^ mice collected at 7 days p.i., a time point at which significant difference in HA response was observed between the WT and C3^-/-^ mice. Injection of WT immune sera to C3^-/-^ mice infected with the virus provided considerable protection (lethality was reduced from 100% to 33%), while injection of C3^-/-^ immune sera failed to provide any protection. These observations ascertain that decreased antibody response in C3^-/-^ mice, at least in part, is responsible for the reduced immune protection in these mice resulting in lethality.

Importance of C3 and C4 in humoral immune responses has been investigated earlier using C3 and C4 deficient mice. These mice showed impaired ability to form germinal centres and develop antigen-specific antibody responses [43]. A similar phenotype was also observed in the C3d receptor (CD21/CD35) knockout mice [44,45]. Importantly, it was also shown that C3 synthesized in the splenic lymphoid compartment is capable of reconstituting the impaired humoral responses in C3^-/-^ mice [46]. The mechanisms involved in C3-mediated germinal center responses include: i) activation of naïve B cells owing to co-ligation of B cell receptor and CD21 (a part of CD19-CD21-CD81 complex) by C3d (a C3 fragment) coupled to the antigen [47,48] and ii) trapping and retention of C3d coupled antigen on follicular dendritic cells expressing CD21/CD35 [49]. The major source of C3 in the splenic lymphoid compartment was thought to be macrophages [46]. In the present study, we show that C3 synthesized by B cells can also partially correct the C3^-/-^ phenotype. These data therefore suggest that apart from macrophages, C3 produced by B cells also play a significant role in initiating the germinal center responses and mounting protective immunity.

Previously it has been shown that C3 deficiency results in enhanced viral spread and impaired recruitment of virus-specific CD4^+^ and CD8^+^ effector T cells in the lung during seasonal influenza infection owing to attenuated T cell priming [16]. Interestingly, such defect was not observed in CD21/CD35-deficient mice suggesting the involvement of other C3 receptor(s) [16]. In a later study, impaired priming of T cells during seasonal influenza virus infection in C3^-/-^ mice was linked to reduced migration of dendritic cells (DCs) from the lung to the draining lymph nodes as a result of lack of direct signaling through C3a and C5a receptors expressed on the lung DCs [27]. In the present study, we observed a high degree of lethality in the WT mice infected with the pandemic influenza virus after blocking the C3aR signaling with little effect after blocking the C5a signaling using receptor antagonists. Similar results were also obtained when we employed C3aR^-/-^ and C5aR^-/-^ mice to study the relative importance of these two receptors during the pandemic influenza virus infection. Thus, our data suggest that primarily the C3aR-mediated signaling is critical for optimum priming of CD4^+^ and CD8^+^ T cells. The CD4^+^ T cells then provide help for optimal generation of neutralizing antibodies, and these antibodies along with CD8^+^ T cell effector function provide protection against the pandemic influenza virus infection.

It is apparent from the above discussion that complement activation and synergy between the various complement pathways is vital for effective control of *in vivo* pandemic influenza virus infection. To illustrate how various complement pathways are triggered during the influenza virus infection and why synergy is critical, we performed a series of *in vitro* experiments. Data obtained revealed a number of features of the virus-complement interactions.

First, the pandemic virus triggered complement only when they were coated by antibodies. No neutralization of the virus was observed by the CP or AP when it was incubated with the sera or plasma alone, but neutralization was apparent by both the pathways when the virus was coated with antibodies. Extrapolation of these results to *in vivo* situation would mean that complement is unable to control the pandemic influenza virus until the appearance of virus-specific antibodies. This proposition gains support from the fact that complement deficient mice do not show any difference in the virus titer compared to the WT mice at 4 days post-infection, a time when antibody response to HA is negligible.

Secondly, the pandemic influenza virus surface is not amenable to C3b deposition. It is well known that deposition of C3b onto the target surface is key to activation of the AP [50], which then culminates in virus neutralization [51]. Direct activation of C3 near the viral surface showed significant deposition of C3b onto the seasonal influenza A(H3N2) virus, but not on the pandemic influenza virus, unless coated by antibodies, which correlated with the neutralization of these viruses. These data thus indicate that modification of the pandemic influenza virus surface by antibodies is necessary for C3b deposition and thereby neutralization. A possible mechanistic explanation for the difference in C3b deposition on the pandemic and the seasonal H3N2 influenza viruses is as below. C3b is deposited onto the activator surface due to its ability to covalently attach to the acceptor molecules on that surface via ester or amide linkages. This attachment of C3b however is not a nonspecific reaction as C3b displays strong preferences for certain carbohydrates and high degree of specificity for particular sites on the acceptor molecules [50,52–54]. For example, Thr144 and Ser1217 have been identified as the major attachment sites for C3b on IgG1 and C4b, respectively [52–54]. HA and NA are the two major surface glycoproteins of the influenza A viruses that project out from its outer surface. It is therefore likely that C3b attachment onto the influenza viruses takes place due to covalent linkage of C3b to HA and/or NA. Since C3b displays specificity for specific residues on the acceptor molecules, it is conceivable that it attaches to specific residues on these molecules. The HA and NA of the pandemic and seasonal H3N2 influenza viruses belong to different antigenic subtypes, therefore, we propose that C3b acceptor site(s) have been altered in the pandemic influenza virus and thus C3b is only attached when its surface is modified by IgG, which is an effective C3b acceptor.

Thirdly, simultaneous activation of the CP and AP support enhanced neutralization of the pandemic virus. Activation of the CP is known to trigger the AP loop resulting in additional deposition of C3b onto the target surface. Here we showed that such amplification of the AP loop as a result of CP activation leads to enhanced neutralization of the pandemic virus. Thus, synergy between the CP and AP result in augmented complement activation ensuing more neutralization of the virus owing to C3b deposition.

**Fig 11:**
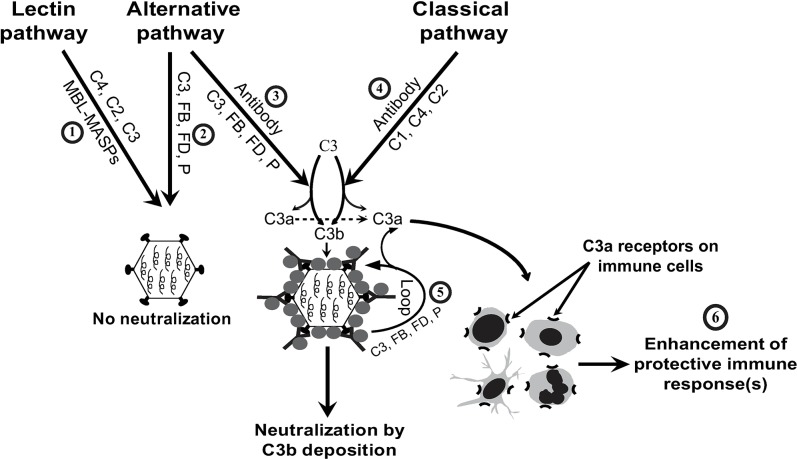
Model for the interplay between CP and AP during A(H1N1)pdm09 virus infection. Synergy between CP and AP is critical to contain the influenza A(H1N1)pdm09 virus infection. 1) The virus is resistant to LP-mediated neutralization (**Fig 9F**). 2) It is also resistant to AP-mediated neutralization (**Figs 8B & 9B**). 3) It becomes susceptible to AP-mediated neutralization when coated with virus-specific antibodies (**Figs 8C & 9C**). 4) It is susceptible to CP-mediated neutralization (**Figs 8A & 9A**). 5) Augmentation of CP initiated complement activation through activation of AP-loop results in enhanced CP-mediated neutralization (**Fig 8D**). 6) Complement activation product C3a helps in the enhancement of protective immune responses owing to activation of immune cells (**Fig 7**).

In summary, our data reveal the importance of cross-talk between the CP and AP that provides sufficient trigger (C3b deposition and C3a production) required for efficient protection against the pandemic influenza virus infection. Based on our present findings and from the earlier studies, we propose the following model for complement-mediated protection during the pandemic influenza H1N1 2009 virus infection **(Fig 11)** wherein: i) recognition of the pandemic influenza virus by antibodies triggers the activation of CP as well as AP leading to C3b deposition and direct neutralization of the virus to a certain extent, ii) the complement activation fragments C3d, as well as C3a generated as a result of complement activation enhance B cell responses and the effector CD4^+^ and CD8^+^ T cell responses, respectively and iii) the effector CD8^+^ T cells and antibodies then efficiently contain the virus infection.

## Materials and methods

### Ethics statement

All the animal experiments performed in this study were approved by the Institutional Animal Ethics Committees of the National Centre for Cell Science, Pune (NCCS) and the National Institute of Virology (NIV), Pune. Use of human serum samples from A(H1N1)pdm09 virus positive and negative individuals for the study was approved by the Institutional Ethical Committees of NCCS and NIV. All adult subjects provided informed written consent; for child participants informed written consent was provided by the parent/guardian of the child.

### Virus

The pandemic influenza A(H1N1)pdm09 virus (A/India/Jln_NIV9436/2009; GenBank accession nos: HM204573, HM241701-07 [12]) and influenza A(H3N2)09 virus (A/India/NIV33041/2009) utilized in this study were isolated in 2009 at NIV, Pune. The virus stocks were prepared by propagating the virus in the embryonated chicken eggs. In brief, the virus was inoculated into the allantoic cavity of 10-day-old chicken embryos. The allantoic fluid was then harvested 72 hr post inoculation and the virus titer were determined by hemagglutination (HA) assay. Samples having HA titers of ≥64 were pooled together to make the virus stock and stored at -80°C in aliquots. The infectious virus titer (50% tissue culture infectious dose, TCID_50_) of the stock was determined as described below and calculated by employing the Reed and Muench method [55]. For ELISA, the virus was inactivated using beta-propiolactone (β–PL) [56] and purified over 10%-50% sucrose gradient.

### Mice

Wild type BALB/c and C57BL/6 mice, C3^-/-^ and C4^-/-^ on C57BL/6 background, and C3aR^-/-^ and C5aR1^-/-^ mice on BALB/c background were purchased from The Jackson Laboratory, USA. Factor B^-/-^ (FB^-/-^) mice on C57BL/6 background and C3^-/-^ mice on BALB/c background were a kind gift from Prof. Marina Botto, Department of Medicine, Imperial College of London. C5-sufficient B10.D2/nSnJ and C5-deficient B10.D2/oSnJ mice were also procured from The Jackson Laboratory, USA. All the mice were bred and housed in a barrier-maintained animal facility of NCCS. Animal experiments were performed in the Biosafety Level 3 (BSL-3) laboratory at NIV, and the mice were housed in HEPA-filtered, negative pressure, individually ventilated cages.

### Cell line, antibodies and sera

Madin-Darby Canine Kidney (MDCK) cell line used for TCID_50_ determination was obtained from the Centers for Disease Control and Prevention, USA and maintained in Dulbecco’s modified Eagles medium (DMEM) containing 10% FCS, 200 U/ml penicillin and 0.2 mg/ml streptomycin at 37°C in a 5% CO_2_ atmosphere with 95% humidity. Human IgG required for the virus neutralization assays was purified from pooled serum samples of A(H1N1)pdm09 virus positive individuals by caprylic acid precipitation method. Mouse IgG needed for the virus neutralization assays was purified from the pooled sera of WT mice infected with the A(H1N1)pdm09 virus using Hi-Trap Protein A column (GE Healthcare Bio-Sciences, Sweden). Homogeneity of the antibodies was assured by SDS-PAGE analysis. Normal human serum (NHS) used as a source of complement was obtained from subjects negative for the A(H1N1)pdm09 antibodies. C1q depleted human serum was purchased from Complement Technologies, USA. Normal (WT), C3^-/-^, C4^-/-^, FB^-/-^ and C5-sufficient and C5-deficient mouse plasma used as a source of complement were obtained from the respective naïve mice by collecting the blood in 20 mM EDTA. Heat-inactivated human serum and mouse plasma were prepared by inactivating them at 56°C for 30 min. Immune mouse serum utilized for rescue of the A(H1N1)pdm09 virus infected C3^-/-^ mice was prepared by drawing the blood from C57BL/6 mice infected with 450 TCID_50_ of the virus at indicated time; serum having HAI antibody titers ≥320 were pooled together and used for the rescue study.

### Virus infection in mice

A group of female mice (~8 week old) were lightly anesthetized and infected i.n. with 450 TCID_50_ of the egg grown A(H1N1)pdm09 virus in 50 μl of allantoic fluid diluted in phosphate-buffered saline (PBS). Mock infection was performed with the allantoic fluid from uninfected eggs diluted in PBS. After infection, the mice were weighed individually each day and observed for signs of illness and mortality. Mice with severe infection and body weight loss of ≥ 30% of their initial body weight were sacrificed; these mice were also considered for calculation of mortality. To determine the virus titers and for histopathology, the mice were sacrificed on days 4 and 7 p.i., and their lungs and blood were harvested under sterile conditions. One part of the lung was utilized for determining the virus titer by TCID_50_ assay, while the other part was fixed in 10% formalin saline for histopathology. Blood was allowed to clot to collect serum for determination of the antibody response using HAI assay and ELISA.

For antagonist studies, wild type mice received daily intraperitoneal injection (i. p.) of either C3a receptor antagonist (C3aRA; SB 290157; GenoMechanix, USA) or C5a receptor antagonist (C5aRA; Ac-Phe-[Orn-Pro-dCha-Trp-Arg]; GenoMechanix, USA) or both (1mg/kg of body weight in 1.16% DMSO) from day -2 to 10 post-infection.

For rescue studies with immune sera, C3^-/-^ mice received two i.p. injections of pooled immune sera (250μl each) at day -1 and 6 post-infection. The immune sera was collected at day 7 post-infection from 20 WT mice infected with A(H1N1)pdm09 virus.

For adoptive transfer studies, C3^-/-^ mice received (i.v.) either total splenocytes (1 x 10^6^ cells/mouse) or purified splenic B or T cells (1 x 10^6^ cells/mouse) isolated from naïve WT or C3^-/-^ mice. After 7 days of adoptive transfer, these mice were infected intranasally with the pandemic influenza virus (450 TCID_50_) and monitored for weight loss and survival. For splenocyte preparation and purification of T and B cells, spleens from WT or C3^-/-^ mice were harvested and minced to prepare single cell suspensions and RBCs were removed using ammonium-chloride-potassium (ACK) lysis buffer. These splenocytes were stained with Alexa Fluor 647 conjugated anti-mouse CD3ε (Clone 145-2C11; Biolegend, San Diego, CA) and FITC conjugated anti-mouse CD19 (clone eBio1D3; eBioscience, San Diego, CA) on ice for 30 minutes. The cells were then washed with PBS and passed through 70 μm pore containing cell strainer. Purification of T cells (CD3ε^+^CD19^-^ cells) and B cells (CD19^+^CD3ε^-^ cells) was performed using BD FACS ARIA III sorter (BD Biosciences, San Jose, CA). The purity of these sorted cells was >96% **(S3 Fig)**.

### Virus titration (TCID_50_) and histopathology

For virus titration, lungs were harvested under sterile conditions, weighed, and homogenized in viral transport medium (Hank’s balanced salt solution containing 250 μg/ml of gentamicin, 2000 U/ml of penicillin and 200 μg/ml streptomycin) to make 10% lung suspension. This suspension was then centrifuged and the supernatant obtained was used for quantitating the virus by TCID_50_ assay. In brief, the lung samples (25 μl) were serially diluted (3-fold) in a 96-well microtiter plate and incubated at 37°C in 5% CO_2_ for 1 hr. Thereafter, 100 μl of MDCK cells (1.5 x 10^4^ cells/well) were added to each well and the plate was kept at 37°C for 18 hrs in 5% CO_2_. The infected cells were then fixed with 80% cold acetone and wells were blocked with 1% milk in PBS-T (PBS containing 0.1% tween 20). To detect viral antigens, wells were washed three times with PBS-T and incubated at 22°C for 1 hr each with 100 μl rabbit anti-influenza A antibody (1:3000 dilution; raised at NIV) followed by a 1:2000 dilution of peroxidase-conjugated goat anti-rabbit IgG antibody (Sigma). Both the above incubation steps were followed by five washes with PBS-T. Finally, the optical density was measured at 490 nm after adding ortho-phenylene diamine (OPD) and stopping the reaction with 1 N H_2_SO_4_. The virus titer (TCID_50_) was calculated using the Reed and Muench method [55].

For histopathology, the lung tissues were fixed in 10% neutral buffered formalin. These tissues were then embedded in paraffin, cut into 4μm thick sections and stained (with hematoxylin and eosin) and analyzed at the Veterinary College Core Facility, Krantisinh Nana Patil College of Veterinary Science, Shirwal.

### Hemagglutination inhibition (HAI) assay

The HAI assay was performed to determine the anti-HA antibody response in the infected mice. In brief, 50 μl of RDE (receptor destroying enzyme; Denka Siken, Japan) treated mice serum (pre-diluted, 1:10) was serially diluted (2-fold) in a V-bottom 96 well microtiter plate (Tarsons, India). The diluted serum was then mixed with 25μl of 8 HA units of β-PL inactivated virus and incubated at room temperature for 30 min. Thereafter, 50μl of 0.5% turkey RBC was added to each well and the plate was kept at 4°C for 30 min before reading. The reciprocal of the last dilution at which the antibodies were still able to inhibit the virus-mediated RBC agglutination was considered as the HAI antibody titer.

### ELISA for virus-specific antibodies

For detection of antibodies against influenza A(H1N1)pdm09 virus in human serum, microtiter plates (Greiner Bio-One) were coated overnight at 4°C with β-PL inactivated virus (250 ng/well). The wells were then blocked with 5% milk, washed thrice and incubated with the diluted sera (1:1600 for IgG; 1:400 for IgM; 1:1600 for IgG1, IgG2, IgG3 and IgG4) for 1 hr at room temperature. To detect the bound IgG antibodies, wells were washed three times with PBS-T and incubated with mouse anti-human IgG, IgG1, IgG2, IgG3 or IgG4 (1:1000 dilution; Sigma Aldrich, USA), followed by rabbit anti-mouse HRP conjugate (1:500 dilution; BioRad) and ABTS. IgM antibodies were detected with goat anti-human IgM HRP (1:250 dilution; Sigma Aldrich, USA). Incubation with class/subclass specific antibodies was for 1 hr at room temperature and that for the conjugate was for 30 min at room temperature; each of the incubation steps, except ABTS, was followed by three washes with PBS-T. The optical density was read at 415 nm.

### ELISA for HA-specific antibodies

Measurement of HA-specific IgM and IgG in sera of A(H1N1)pdm09 virus infected mice was performed as below. The microtiter plates (Greiner) were coated with pandemic influenza virus HA protein [200 ng/well; rHA of the H1N1 (A/California/7/2009); ThermoFisher Scientific, Waltham, MA] by keeping the plates overnight at 4°C; control wells were coated with similar amount of BSA. The wells were then blocked by adding 5% milk, washed once with PBS-T and incubated with 1:50 diluted heat inactivated mouse sera for 1 hr at room temperature. Thereafter, the wells were washed three times with PBS-T and incubated with goat anti-mouse IgM HRP conjugate (1:1000 dilution; Sigma Aldrich) or rabbit anti-mouse IgG HRP conjugate (1:1000 dilution; Sigma Aldrich) for 30 min at room temperature. The unbound conjugate was then washed, ABTS was added, and optical density was measured at 415 nm. Mouse sera and conjugates were diluted in PBS-T containing 0.5% milk and 0.5% BSA.

### Virus neutralization assay

Virus neutralization was studied using the human as well as the mouse complement. To determine the human CP-mediated neutralization of the virus, 32 μl of the A(H1N1)pdm09 virus (500 TCID_50_) was mixed with 12 μl of human IgG (36 μg) purified from influenza A(H1N1)pdm09 virus positive subjects and 16 μl or 32 μl serum from the pandemic virus negative individuals as a source of complement in the presence of 0.15 mM Ca^++^ and 0.5 mM Mg^++^ in a total volume of 320 μl DMEM containing 1% BSA. The reaction mixture was then incubated at 37°C for 1 hr, diluted in DMEM, and assayed for virus titer by determining TCID_50_. For the human AP-mediated neutralization of the virus, 70 μl of the A(H1N1)pdm09 virus (850 TCID_50_) was mixed with 70 μl, 210 μl or 350 μl of serum from the pandemic virus negative individuals as a source of complement in the presence of 5 mM Mg-EGTA in a total volume of 700 μl DMEM containing 1% BSA. As for CP, the reaction mixture was then incubated at 37°C for 1 hr, diluted in DMEM, and assayed for virus titer by determining TCID_50_. Virus neutralization by the mouse CP and AP of complement was performed in a manner essentially similar to that described above for the human CP and AP with the exception that indicated amount of mouse plasma instead of sera was used as a source of complement and excess of Ca^++^/Mg^++^ and Mg^++^ was added to neutralize EDTA present in the plasma for measuring CP- and AP-mediated neutralization, respectively.

For the AP loop studies, CP-mediated neutralization was performed with FB^-/-^ mice plasma. To study the role of antibodies in AP activation, virus was first incubated with virus specific antibodies followed by AP-mediated neutralization with human serum and mice plasma respectively, as mentioned.

### C3b deposition assay

ELISA plates were coated with 300 ng/well of virus [A(H1N1)pdm09 or A(H3N2] overnight at 4°C in PBS. The wells were then blocked with 5% skimmed milk, washed once, and reactions were set up on ice by adding three different concentrations of AP components (Rxn#1: 6 μg C3, 1.5 μg FB and 60 ng FD; Rxn#2: 4 μg C3, 1 μg FB and 40 ng FD; Rxn#3: 2 μg C3, 0.5 μg FB and 20 ng FD) in the presence (5 μg, 2.5 μg or 1.25 μg) or absence of antibody in a total volume of 100 μl GVB containing 5 mM Mg-EGTA. Thereafter, the plate was incubated at 30°C for 30 min and washed three times. C3b deposited on the viral surface was detected by adding anti-human C3 HRP conjugate (1:1000 dilution, ICN; 45 min incubation at room temperature) followed by ABTS. The optical density was read at 415 nm.

### Screening of A(H1N1)pdm09 virus positive human serum for complement binding antibodies

A total of 21 human serum samples from the A(H1N1)pdm09 virus positive individuals were screened for the presence of complement binding antibodies. In brief, sera of the infected individuals were heat inactivated at 56°C for 30 min and utilized for virus neutralization in the presence or absence of human complement. For neutralization, 850 TCID_50_ of the A(H1N1)pdm09 virus in 70 μl was incubated with 10 μl of heat inactivated serum from the virus positive subject and 35 μl of virus negative human serum as a source of complement in the presence of 0.15 mM Ca^++^ and 0.5 mM Mg^++^ in a total volume of 700 μl at 37°C for 1 hr. The reaction mixture was then titrated for infectious virus by TCID_50_ as described above.

### Statistical analyses

One-way ANOVA followed by Tukey’s Post-Hoc test was performed to compare different groups. Comparison between two groups was made using Mann-Whitney Rank Sum test. The percent survival of mice in different groups was plotted as Kaplan-Meier plots and analyzed using Log-Rank test. Results of *in vivo* studies are presented as mean ± SEM, while results of *in vitro* studies are presented as mean ± SD. All the statistical analyses were performed using PASW Statistics 18 software (New York, USA).

## Supporting information

S1 Fig Histopathological changes in mock infected lungs at day 4 and 7 post challenge. WT and complement deficient mice (C3^-/-^, C4^-/-^ and FB^-/-^ on C57BL/6 background) were challenged intranasally with the normal allantoic fluid diluted in PBS, euthanized at day 4 and 7 post mock-infection, and lungs were collected for histopathological analysis. Sections shown are representative (n = 6). All the tissue section showed normal lung architecture with intact lung alveoli (filled arrowhead), bronchial epithelium (filled arrow) and vascular endothelium linings (unfilled arrowhead). Magnification = 100X.
(PDF)

S2 Fig IgG1 response is predominant during A(H1N1)pdm09 virus infection and correlate with the ability of sera to neutralize the virus in the presence of complement. A total of 21 A(H1N1)pdm09 positive human sera were assayed by ELISA to determine the virus-specific antibody response generated during the infection. The A_415_ values are shown for comparison of antibody levels within the subclass of immunoglobulins. A) IgG and IgM response. B) IgG subclass response. C) Correlation between virus-specific antibody levels in the sera and its ability to neutralize in the presence of complement (TCID_50_). Significant correlation was observed only between neutralization potential of sera in the presence of complement and IgG1 (r = -0.628; p < 0.002). Correlation coefficient (r) was calculated using Pearson product moment correlation. Correlation between IgG1 and TCID_50_ was shown again for comparison.
(PDF)

S3 Fig Purity of T and B cells. Spleens were harvested from WT and C3^-/-^ mice on C57BL/6 background (n = 4 mice/group) and minced to prepare single cell suspensions. RBCs were removed using ammonium-chloride-potassium (ACK) lysis buffer. Splenocytes were then washed with PBS and stained with Alexa Fluor 647 conjugated anti-mouse CD3ε and FITC conjugated anti-mouse CD19 monoclonal antibodies. T cells (CD19^-^CD3ε^+^ cells) and B cells (CD19^+^CD3ε^-^ cells) were sorted using BD FACS ARIA III sorter. The purity of sorted B and T cells were analyzed and plotted as dot plots.
(PDF)
