# Supplementary material for: Synergy between the classical and alternative pathways of complement is essential for conferring effective protection against the pandemic influenza A(H1N1) 2009 virus infection
Source: PLoS Pathog. 2017 Mar 16;13(3):e1006248. doi: 10.1371/journal.ppat.1006248 (PMC5354441; doi:10.1371/journal.ppat.1006248)
Supplement: S1 Fig — WT and complement deficient mice (C3-/-, C4-/- and FB-/- on C57BL/6 background) were challenged intranasally with the normal allantoic fluid diluted in PBS, euthanized at day 4 and 7 post mock-infection, and lungs were collected for histopathological analysis. Sections shown are representative (n = 6). All the tissue section showed normal lung architecture with intact lung alveoli (filled arrowhead), bronchial epithelium (filled arrow) and vascular endothelium linings (unfilled arrowhead). Magnification = 100X. (PDF) [file ppat.1006248.s001.pdf]

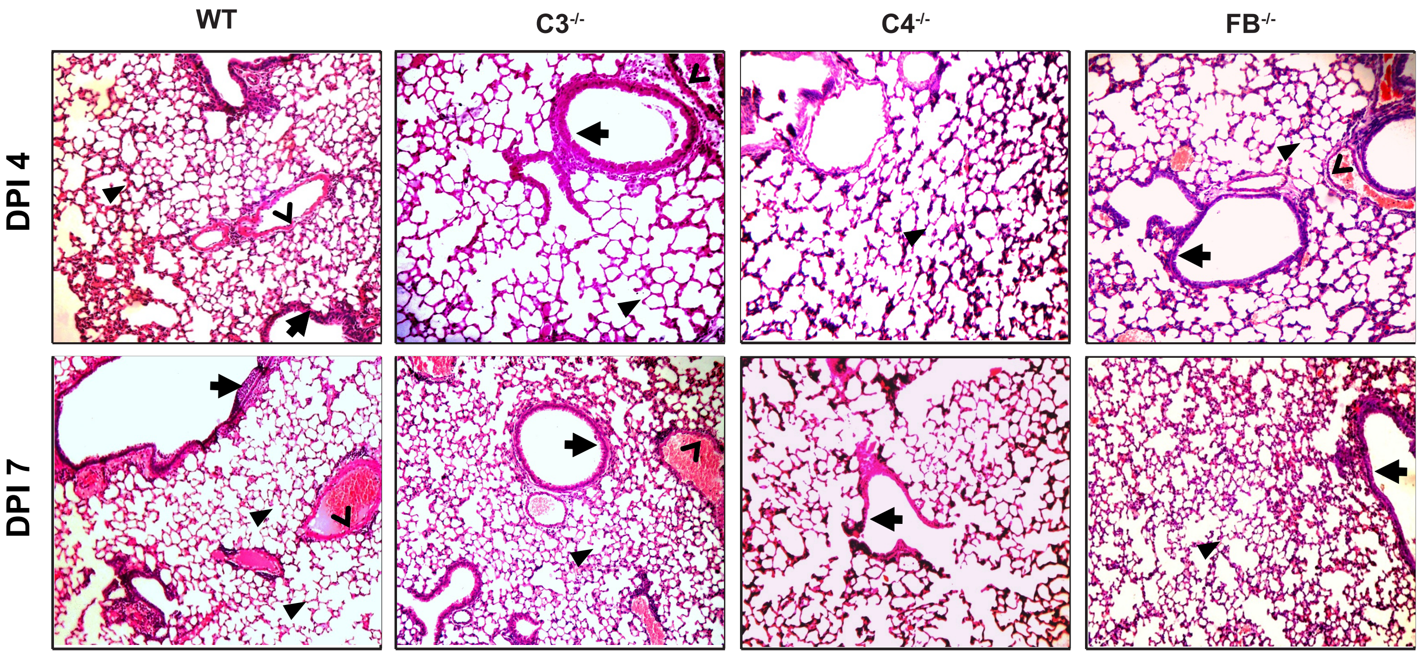

Fig. S1: Histopathological changes in mock infected lungs at day 4 and 7 post challenge. WT and complement deficient mice (C3$^{-/-}$, C4$^{-/-}$ and FB$^{-/-}$) were challenged intranasally with the normal allantoic fluid diluted in PBS, euthanized at day 4 and 7 post mock-infection, and lungs were collected for histopathological analysis. Sections shown are representative (n = 6). All the tissue sections showed normal lung architecture with intact lung alveoli (filled arrowhead), bronchial epithelium (filled arrow) and vascular endothelium linings (unfilled arrowhead). Magnification = 100X.
