# Supplementary figures and images for: Synergy between the classical and alternative pathways of complement is essential for conferring effective protection against the pandemic influenza A(H1N1) 2009 virus infection
Source: PLoS Pathog. 2017 Mar 16;13(3):e1006248. doi: 10.1371/journal.ppat.1006248 (PMC5354441; doi:10.1371/journal.ppat.1006248)

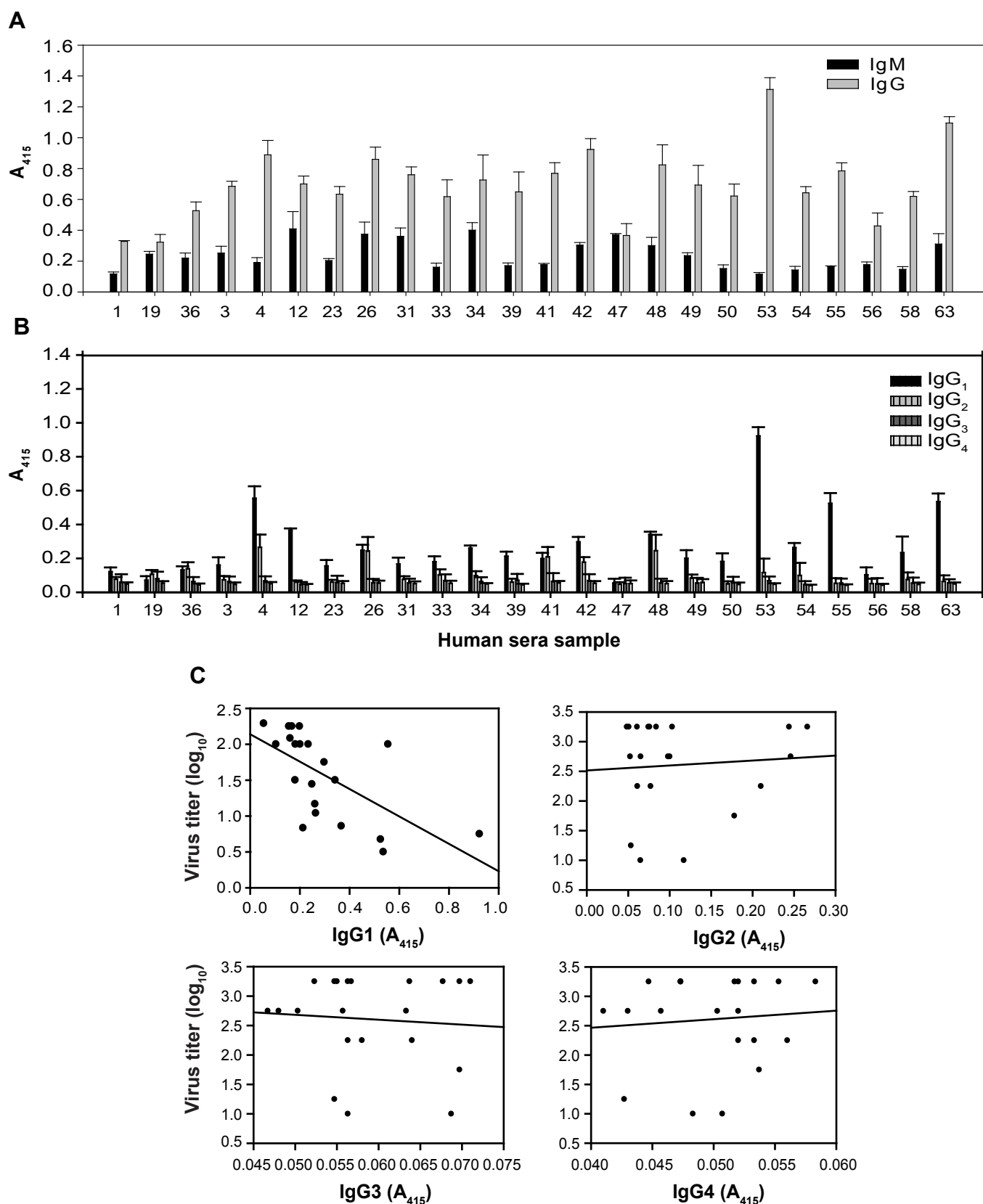

Supplement: S2 Fig — A total of 21 A(H1N1)pdm09 positive human sera were assayed by ELISA to determine the virus-specific antibody response generated during the infection. The A415 values are shown for comparison of antibody levels within the subclass of immunoglobulins. A) IgG and IgM response. B) IgG subclass response. C) Correlation between virus-specific antibody levels in the sera and its ability to neutralize in the presence of complement (TCID50). Significant correlation was observed only between neutralization potential of sera in the presence of complement and IgG1 (r = -0.628; p < 0.002). Correlation coefficient (r) was calculated using Pearson product moment correlation. Correlation between IgG1 and TCID50 was shown again for comparison. (PDF) [file ppat.1006248.s002.pdf]
