# Supplementary material for: Synergy between the classical and alternative pathways of complement is essential for conferring effective protection against the pandemic influenza A(H1N1) 2009 virus infection
Source: PLoS Pathog. 2017 Mar 16;13(3):e1006248. doi: 10.1371/journal.ppat.1006248 (PMC5354441; doi:10.1371/journal.ppat.1006248)
Supplement: S3 Fig — Spleens were harvested from WT and C3-/- mice on C57BL/6 background (n = 4 mice/group) and minced to prepare single cell suspensions. RBCs were removed using ammonium-chloride-potassium (ACK) lysis buffer. Splenocytes were then washed with PBS and stained with Alexa Fluor 647 conjugated anti-mouse CD3ε and FITC conjugated anti-mouse CD19 monoclonal antibodies. T cells (CD19-CD3ε+ cells) and B cells (CD19+CD3ε- cells) were sorted using BD FACS ARIA III sorter. The purity of sorted B and T cells were analyzed and plotted as dot plots. (PDF) [file ppat.1006248.s003.pdf]

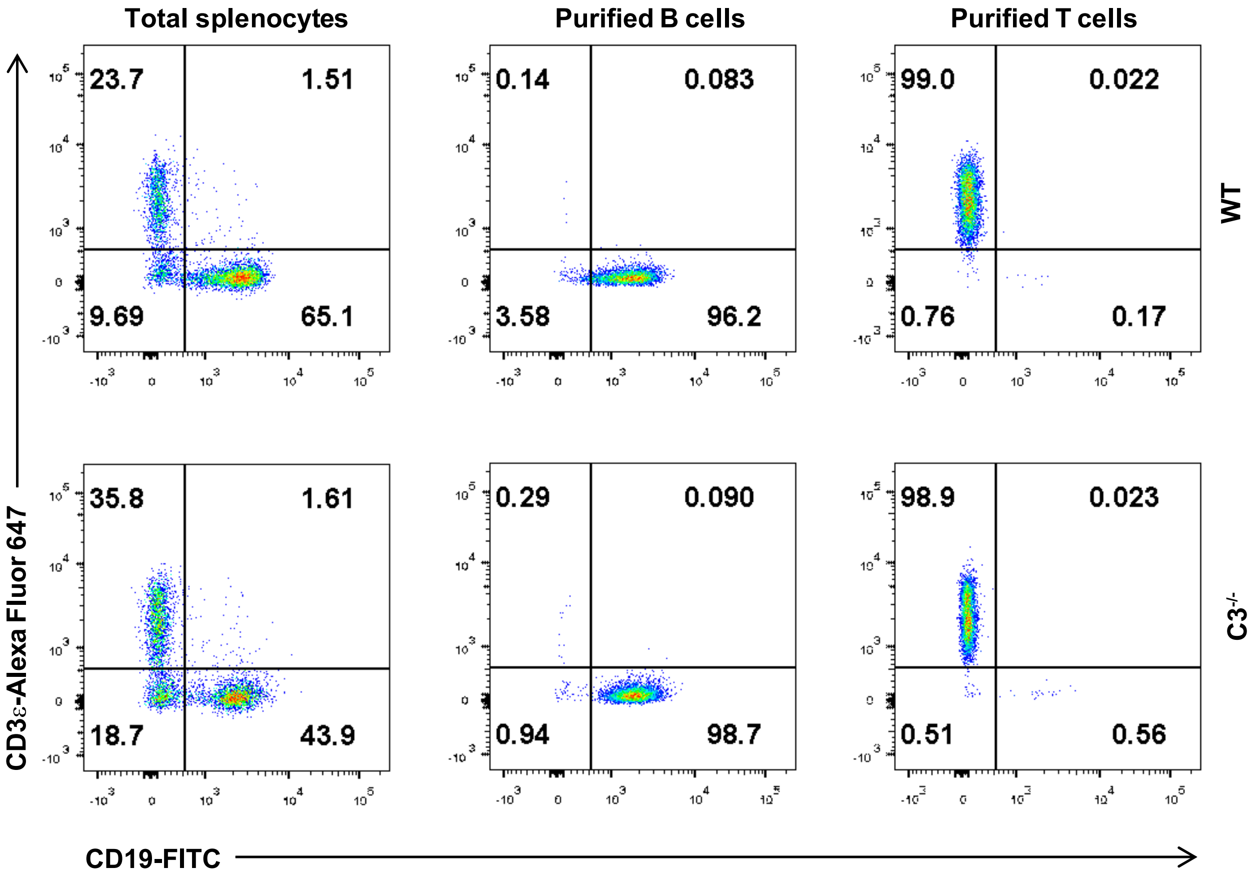

Fig. S3: Purity of T and B cells.

Spleens were harvested from WT and $C3^{-/-}$ mice (n= 4 mice/group) and minced to prepare single cell suspensions. RBCs were removed using ammonium-chloride-potassium (ACK) lysis buffer. Splenocytes were then washed with PBS and stained with Alexa Fluor 647 conjugated anti-mouse CD3ε and FITC conjugated anti-mouse CD19 monoclonal antibodies. T cells ($CD19^{-}CD3\varepsilon^{+}$ cells) and B cells ($CD19^{+}CD3\varepsilon^{-}$ cells) were sorted using BD FACS ARIA III sorter. The purity of sorted B and T cells were analyzed and plotted as dot plots.
